# Analysis of Prognostic Factors in High-Grade Osteosarcoma of the Extremities in Children: A 15-Year Single-Institution Experience

**DOI:** 10.3389/fonc.2016.00022

**Published:** 2016-02-02

**Authors:** Liliana Vasquez, Fanny Tarrillo, Monica Oscanoa, Ivan Maza, Jenny Geronimo, Gloria Paredes, Jose María Silva, Luis Sialer

**Affiliations:** ^1^Division of Pediatric Oncology, Rebagliati Hospital, Lima, Peru; ^2^Department of Orthopedics, Rebagliati Hospital, Lima, Peru

**Keywords:** osteosarcoma, children, prognostic factors

## Abstract

**Background:**

The aim of this retrospective study was to define clinical and pathological features and prognostic factors among children and adolescents diagnosed with high-grade osteosarcoma of the extremities.

**Methods:**

A total of 73 patients younger than 18 years diagnosed with primary osteosarcoma of the extremities between January 1998 and December 2013 were retrospectively evaluated. Prognostic factors, such as age, gender, primary tumor site, alkaline phosphatase and lactate dehydrogenase levels, metastatic disease, pathological fracture, histological response, and surgery type, were analyzed to evaluate their effects on overall survival (OS) and event-free survival (EFS).

**Results:**

At a median follow-up of 30 months (1.5–152), OS and EFS at 5 years were 64.5 ± 8.1 and 48.5 ± 8.7% for patients with localized disease; and 16.2 ± 7.9 and 14.4 ± 7.3% for patients with initial metastatic disease, respectively. In patients with localized disease, conservative surgery was performed on 22 of 46 patients (43.5%), and there was no significant difference in survival rates among patients who had conservative vs. radical surgery (*p* = 0.65). Although tumor size (>12 cm) was significant prognostic factor in univariate analysis; multivariate analysis identified elevated levels of alkaline phosphatase (*p* = 0.033) and poor response to neoadjuvant chemotherapy (*p* < 0.001) only as independent prognostic factors. Age, histological type, pathological fracture, and primary tumor site did not significantly affect prognosis.

**Conclusion:**

Initial elevated presence of alkaline phosphatase in serum and poor histological response after neoadjuvant chemotherapy were significant factors for unfavorable prognosis. It is necessary to optimize staging and treatment intensification to improve survival rates, especially among patients with metastasis at initial presentation.

## Introduction

Osteosarcoma is the most frequent malignant bone tumor at pediatric age. Surgical removal of the tumor remains the most important treatment; however, survival rates have increased due to the advent of chemotherapy (1–3). In prospective studies, 5-year event-free survival (EFS) rates between 55 and 75% have been reported (4–6), and recurrence occur approximately 30–40% of patients with localized disease, in spite of complete surgical resection of primary tumor and an intense regimen of chemotherapy (7). These findings reveal an important need to clarify prognostic factors for recurrence or poor survival.

Currently, several studies have already identified clinical or pathological features associated with worse outcome. Some published data contain a reduced but homogeneous number of cases of limited statistical significance (8, 9). Moreover, multicenter research studies differ in data collection and therapeutical schemes (10–13), which makes it difficult to compare and reveals contradictory results.

In our region, there are no published studies defining prognostic factors for osteosarcoma, which is essential to improve the development of new and adapted strategies according to risk groups so as to improve survival rates.

The aim of the present study is to define clinical and pathological features and prognostic factors related to survival rates among pediatric patients diagnosed with high-grade osteosarcoma of the extremities in our country.

## Materials and Methods

### Patients

Seventy-three patients diagnosed with primary high-grade osteosarcoma of the extremities (both localized and metastatic) were retrospectively evaluated. All patients were treated at the Pediatric Oncology Unit of the Rebagliati Hospital in Lima, Peru between January 1998 and December 2013. All patients who received chemotherapy and surgical treatment in our hospital were included.

### Diagnostic Methods and Staging

Plain X-rays and CT scans or MRI of the primary tumor were performed at the time of diagnosis, prior to surgery, and at the end of therapy. Chest CT scan and bone scintigraphy were performed to detect distant metastases. All patients underwent confirmatory biopsy (open or by tru-cut needle) in our institution, and the diagnosis was confirmed by conventional light microscopy (histology). All cases of high-grade osteosarcoma were included (conventional, telangiectatic, fibroblastic, and small cell subtype). Patients with low grade tumors, such as parosteal osteosarcoma, were excluded.

### Systemic Treatment

After diagnosis and staging, the patient started neoadjuvant chemotherapy. Seventy-one patients (97.3%) received chemotherapy based on SEOP-95 consisted of high-dose methotrexate at 12 g/m^2^ per cycle with leucovorin rescue for 11 cycles, adriamycin at 75 mg/m^2^ per cycle for 6 cycles, cisplatin at 120 mg/m^2^ per cycle for 2 cycles. Additionally, ifosfamide at 9 g/m^2^ was used for six cycles. Length of chemotherapy treatment was 37 weeks. Definitive surgery was scheduled on week 15. A chemotherapy regimen without methotrexate (OS99) was used in two patients due to renal toxicity secondary to methotrexate (after two cycles on the previous regimen). Neoadjuvant chemotherapy treatment was lengthened (*n* = 4) or shortened (*n* = 2) in six patients (8.3%) due to problems related to social security coverage or availability of prosthesis.

### Local Treatment

The first definitive surgical treatment was documented as conservative (resection of primary tumor) with or without endoprosthesis (*n* = 31); or radical (amputation or disarticulation) (*n* = 42). Surgery type and reconstruction were decided according to tumor site and extension, patient’s age, and presence of compromised neurovascular structures.

### Statistical Analysis

Data about time from first symptoms to beginning of therapy, age, gender, primary tumor site, initial alkaline phosphate (AP) and lactate dehydrogenase (LDH) levels, metastatic disease at onset, presence of pathological fractures, histological response or necrosis after completion of neoadjuvant chemotherapy (<90% and more or equal to 90%) (14, 15), and type of surgery were evaluated. SPSS 22.0 Statistics was used (SPSS Corp., USA). Overall survival (OS) was defined as the time from diagnosis to death from any cause and EFS was defined as the time from diagnosis to treatment failure, secondary neoplasm, or death, whichever came first. Patients who did not suffer an event were sensored at the time of last follow-up. OS and EFS rates were analyzed, according to Kaplan–Meier curves. A multivariate Cox proportional hazards regression analysis was performed to determine which parameters were significant. A 95% confidence interval level was used; being a *p* < 0.05 was considered significant.

### Ethical Considerations

Our study was not set up as a study or research project; hence, we did not seek informed consent or ethical committee approval due to it does not report on primary research. Absolutely, all data analyzed were collected as part of routine diagnosis and treatment, and all patients were diagnosed and treated according to institutional guidelines and agreements. All laboratory tests (as well as recording all other variables included in our analysis) are essential for confirming diagnosis and classifying patients, and they are done for each patient without fail and as part of routine care. Moreover, this paper does not report on the use of experimental therapies. We looked retrospectively at outcomes for a long-term cohort of patients treated as a process of an audit/evaluation, so as to improve our quality of care.

## Results

### Patient Characteristics

A total of 73 patients under 18 years of age were included. Patient characteristics are listed in Table 1. The mean age at diagnosis was 14 years (range, 5–17 years). Sixty-six percent of patients were male (45 patients). Average length of follow-up period was 30 months (1.5–152 months). There was metastatic disease at initial presentation in 27 patients (37%), being the lungs the most commonly affected organ (24 patients), followed by bone metastases (3 patients). Latency time from manifestation of the first symptoms and the beginning of treatment was 4.3 months on average, with a range of 0.3–19 months.

**Table 1:** **Demographic and clinical characteristics (*N* = 73)**.

Characteristic	Number of patients (%)
Male (*n*, %)	45 (61.6%)
Age at diagnosis (years)	
Mean	14
Range	5–17
Histological subtype	
Osteoblastic	52 (71.3%)
Chondroblastic	12 (16.4%)
Telangiectatic	5 (6.8%)
Fibroblastic	3 (4.1%)
Other	1 (1.4%)
Location of tumor	
Distal femur	33 (45.2%)
Proximal tibia	15 (20.5%)
Proximal femur	10 (13.7%)
Humerus	9 (12.3%)
Fibula	6 (8.3%)
Distant metastases at onset	
Yes	27 (37%)
No	46 (63%)
Histological response (*n* = 40)	
≥90%	21 (52.5%)
<90%	19 (47.5%)
Elevated serum AP	58 (79.5%)
Elevated serum LDH	57 (78.1%)
Type of surgery	
Conservative	31 (42.5%)
Amputation	42 (57.5%)
Tumor size (*n* = 32)	
≤12 cm	25 (78.1%)
>12 cm	7 (21.9%)

In the localized group, conservative surgery was performed in 22 out of 46 patients (47.8%), and no significant difference was observed in survival rates related to type of surgery (*p* = 0.65). In both groups (localized and metastatic), radical surgery (amputation or disarticulation) was performed in 42 cases, and limb-preservation surgery (with or without endoprosthesis) was done on 31 patients. Patients with localized disease had better chances of having conservative surgery compared to those with metastatic disease at presentation (*p* = 0.003) (47.8 vs. 25.9%).

Twenty-three patients (31.5%) had recurrent disease (3 local and 20 metastatic). In 19 cases (82.6%), recurrence occurred within the first 3 years of diagnosis. Initial treatment was surgical if recurrence was local (three patients), with amputation in two cases and tumor resection in one patient. These patients received second-line chemotherapy (etoposide or carboplatin based). Only the patient who had tumor resection surgery had no evidence of disease for 42 months, whereas the other two died from progressive disease. Treatment in case of metastatic recurrence (20 patients) was surgical in 13 cases (65%) (lung metastasectomy, bilateral, when possible) or second-line systemic chemotherapy alone in 7 cases (35%) in case surgery was not possible (due to irresectable tumor or multiple lung nodules), presence of distant lesions in the liver and brain, or refusal to surgery. Nine of 20 patients (45%) died from progressive disease. In two cases of metastatic pulmonary relapse, after surgical resection of lung metastases, high-dose chemotherapy was administered and autologous stem cell transplant was performed. In one of those two cases, the patient died from progressive of disease, and the other had a complete remission with a follow-up of 6 years.

### Prognostic Factors for Overall Survival

Univariate and multivariate analysis of factors predicting treatment failure is shown in Table 2. An initial elevated serum level of AP (*p* = 0.027), poor histological response (necrosis of <90%) (*p* < 0.01), large tumor size (>12 cm) (*p* < 0.01), and metastatic disease (*p* < 0.01) was statistically significant for worse survival. All these variables except tumor size remained significant on multivariate analysis.

**Table 2:** **Summary of univariate Cox proportional hazards model for overall survival**.

Variables	Univariate LRT	HR (95% CI)	Multivariate LRT	HR (95% CI)
	*p*-Values		*p*-Values	
Male gender	0.78	1.14 (0.44–2.94)		
Age, <10 years	0.09	3.73 (0.42–2.47)		
Histological subtype	0.96	1.02 (0.15–6.64)		
Location of tumor	0.57	0.77 (0.31–1.94)		
Initial metastases	**<0.01**	4.88 (3.48–40.57)	**<0.01**	4.6 (3.71–38.93)
Necrosis >90%	**<0.01**	0.08 (0.02–0.31)	**<0.01**	0.13 (0.06–0.28)
Tumor size >12 cm	**<0.01**	4.01 (2.43–8.12)	0.058	3.5 (0.92–9.39)
Initial raised ALP	**0.027**	3.90 (1.11–13.71)	**0.033**	3.1 (1.10–5.27)
Initial raised LDH	0.18	2.13 (0.63–6. 67)		
Pathological fracture	0.30	1.86 (0.56–6.22)		

*LRT, likelihood ratio test; HR, hazards ratio; CI, confidence interval*.

*Significant variables (*p* values <0.05) are in bold*.

Gender, age, initial LDH levels, presence of pathological fracture, histological subtype (osteoblastic, chondroblastic, telangiectatic or fibroblastic), latency time of symptoms, primary site tumor, and type of surgery were not significant for survival in univariate or multivariate analysis.

Surgical margins were compromised in two patients (2.8%) who had metastasis at initial presentation and underwent limb amputation after neoadjuvant chemotherapy. Both died due to progressive disease. No statistical analysis was performed due to small number of patients.

Some patients had an alteration in the neoadjuvant chemotherapy sequence (it was either longer or shorter) due to problems with health insurance or with availability of prosthesis. There was no statistical difference with respect to those who received standard therapy.

### OS and EFS

**Figure 1:**
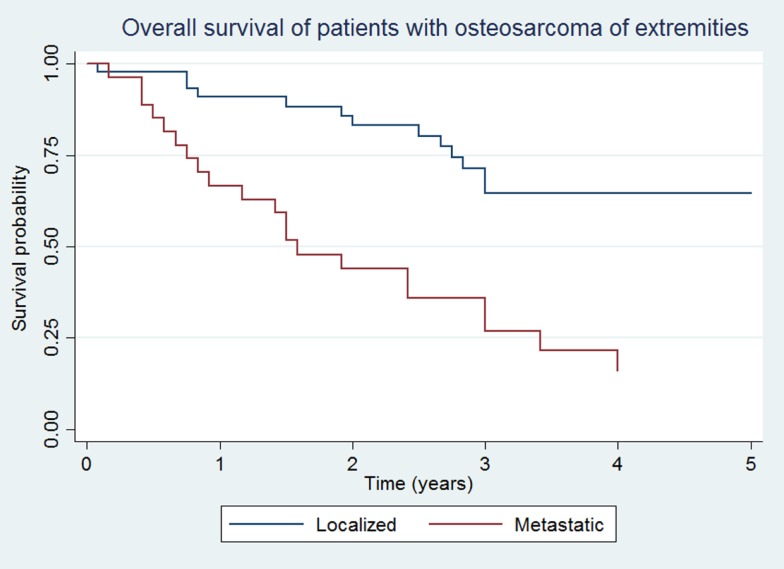
**Overall survival of patients with localized and metastatic osteosarcoma of extremities (*N* = 76)**.

**Figure 2:**
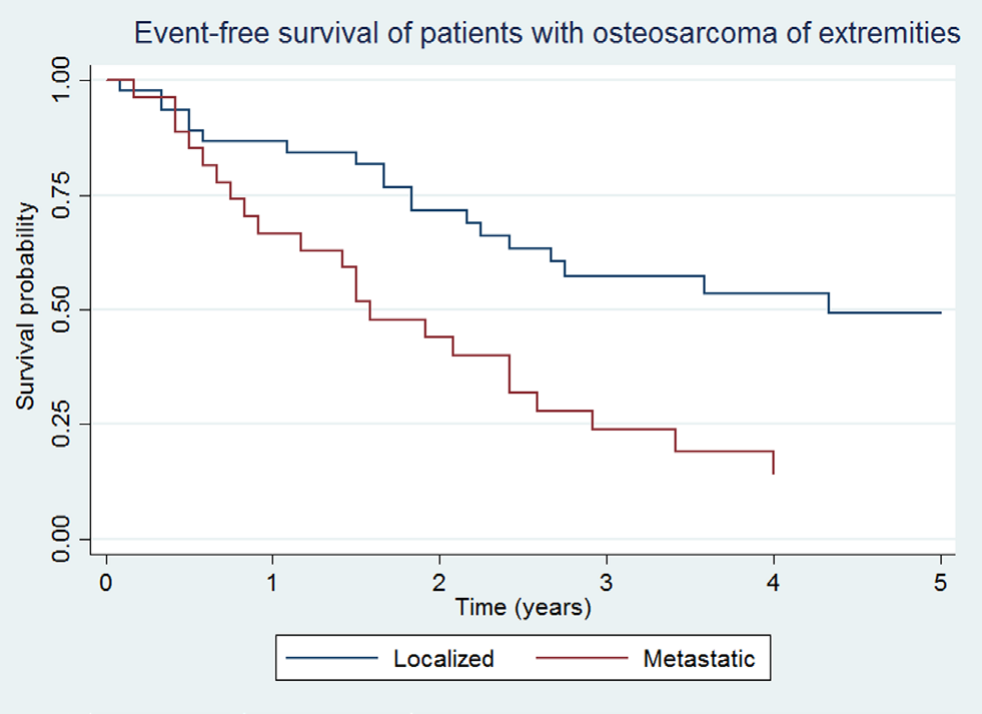
**Event-free survival of patients with localized and metastatic osteosarcoma of extremities (*N* = 76)**.

Overall survival and EFS at 5 years was 64.5 ± 8.1 and 48.5 ± 8.7% for the localized group, and 16.2 ± 7.9 and 14.4 ± 7.3% for the metastatic group, respectively (Figures 1 and 2).

## Discussion

Identifying prognostic factors in osteosarcoma patients is vital to define risk groups. A number of clinical and pathological variables, such as histological subtype, age, gender, elevated levels of AP or LDH, and genetic variations have been previously studied with prognostic significance, but often with contradictory results due to lack of homogeneity in analysis and methods. In a systematic revision of prognostic factors for osteosarcoma, it was found that histological response, presence of metastasis, and primary tumor size and site were important prognostic factors (16).

Our study found considerably higher incidence of clinically detectable metastatic disease at initial presentation when compared to expected rates in developed countries (10–20%) (17–19), or even when compared to other Latin American studies, like those done in Brazil (20.8%) (5, 20, 21), being almost 40%. This is probably due to delay in diagnosis and advanced disease at the moment of diagnosis, and it turns out to be the first cause of mortality among these patients, which makes it a significant prognostic factor, as reported by prior studies (5, 6).

As previously described (17), presence of pathological fractures ranges from 7 to 18% (in our study 14%) and, while it did not proof to be prognostic as reported earlier (22), it is an important indicator of late diagnosis in our cases.

Age has been identified as a prognostic factor for osteosarcoma. Patients under the age of 10 (18) or 12 years (9) have poorer survival rates in some reports, which suggest aggressive behavior of this disease in small children. Nevertheless, more recent studies have failed to confirm the latter (13, 19), and we have also not observed this in our study. In a recent study, patients with age above 40 years also carried a dismal prognosis (23).

Gender was not prognostic, as has already been mentioned in European (13, 18) and American report studies (9). However, Brazilian (20) and Scandinavian (24) studies have included observations about female gender having a better prognosis.

The present study has not found any significant difference in survival rates according to histological subtype, as previously reported (25). According to other authors, there is evidence of lower risk of recurrence in fibroblastic and telangiectatic subtypes (26). It has also previously been mentioned that the chondroblastic subtype could be the one with the best prognosis (27). Furthermore, histological response has been more favorable in fibroblastic and telangiectatic groups, and less favorable in the chondroblastic group (28).

Prior studies have revealed that surgery type (radical vs. conservative) in patients with high-grade non-metastatic osteosarcoma does not affect survival or local recurrence rates (29–31). This is supported by our study, where no significant difference was found in both groups. In a study conducted by Bacci et al., 560 non-metastatic osteosarcoma patients were examined, and no variation in survival rates was found when considering surgery types. The percentage of conservative surgery, even though it has increased over the years and is currently at 20% of total operations, is still lower than the rates published by other groups (5).

Degree of necrosis as histological response to neoadjuvant chemotherapy is currently considered the most important prognostic factor in patients with high-grade osteosarcoma, according to reports in previous studies (5–7, 32). The percentage of patients who have a favorable response (≥90%) goes from 50 to 60% (32), whereas, it was 52.5% in our study.

The prognostic value of AP in osteosarcoma has previously been reported (9, 31, 32) and has been confirmed in our study. Bacci et al. have reported that initial values of more than four times the normal level are linked to lower EFS rates (33).

Event-free survival rates among osteosarcoma patients worldwide are between 55 and 75%, which has not improved significantly in recent years. Studies in developed countries mention survival rates of 70–75% in localized disease (4, 6, 34, 35). A study by the European group (COOS) done on 1702 patients found that there was 5-year OS of 63.3% and an EFS of 52.8% (34). A study from the Rizzoli Institute describes patients with localized disease at initial presentation with 10-year overall and EFS of 70 and 59%, respectively (4).

Survival rates are considerably lower in metastatic disease, having an average of 10–40% patients alive long term. Meyers et al. (36) reported that only 11% of 64 patients survived, with an average length of 20 months. Kager et al. (37) described a 5-year overall and EFS in 202 patients of 29 and 18%, respectively. Similar reports have been done by European groups with 16% rates (*n* = 45) and by American groups with 53% rates (*n* = 30), the latter study having important differences, such as a higher proportion of patients with unique lung metastases and excluding patients with irresectable disease (38, 39). In Peru, there are no previous reports about survival in osteosarcoma patients. Our results showed overall and EFS rates comparable to studies in developing countries (40–42).

The present study has its main limitation on the small number of patients. Similarly, there is missing information in some of the cases, due to its retrospective design, which encourage us to standardize and improve clinical records in our institution.

Nevertheless, its main strength is that it represents a first analysis of its kind in the medical literature of our country and blazes a trail for more ambitious and wider ranging future studies.

## Conclusion

The presence of initial serum elevated AP levels and a poor histological response after neoadjuvant chemotherapy were significant predictors in children with osteosarcoma of extremities. Therefore, it reveals the need for cooperative studies that outline strategies on the basis of risk factors. It is necessary to optimize staging and intensification of treatment to improve survival rates, especially among patients with metastases at initial presentation.

## Author Contributions

FT, MO, and GP actively contributed to collect data and statistical process. JG, LS, and JS have reviewed and made corrections to the final version of the manuscript. All authors made important contributions to the conception or design and analysis of the work; revising it critically and made a final approval of the version to be published with agreement with the accuracy and integrity of the information on the manuscript.

## Conflict of Interest Statement

The authors declare that the research was conducted in the absence of any commercial or financial relationships that could be construed as a potential conflict of interest.
